# Functionalized Nanoparticles: A Paradigm Shift in Regenerative Endodontic Procedures

**DOI:** 10.7759/cureus.32678

**Published:** 2022-12-19

**Authors:** Vinoo Subramaniam Ramachandran, Mensudar Radhakrishnan, Malathi Balaraman Ravindrran, Venkatesh Alagarsamy, Gowri Shankar Palanisamy

**Affiliations:** 1 Department of Conservative Dentistry and Endodontics, Bharath Institute of Higher Education and Research, Chennai, IND; 2 Department of Conservative Dentistry and Endodontics, Sree Balaji Dental College & Hospital, Chennai, IND; 3 Department of Microbiology, Sathyabama Institute of Science and Technology, Chennai, IND; 4 Department of Oral Pathology and Microbiology, RVS Dental College and Hospital, Chennai, IND

**Keywords:** nanoscaffolds, regenerative endodontics, nanofiber, nanoparticles, nanotechnology

## Abstract

Clinical treatment of inflamed tooth pulp mostly involves the removal of the entire pulp tissue. Because the vitality of the tooth is important for its ability to function, optimal regenerative biomaterials must be developed to maintain the vitality. Despite vast advances in the field of endodontics, the clinical translation of regenerative endodontic procedures and materials remains challenging. Patient-specific, tissue-derived stem cells play a major role in regeneration and revascularization, and these stem cells require an infection-free environment for a successful outcome. However, the high doses of antibiotics currently used to maintain an infection-free environment for tissue regeneration can be toxic for the stem cells. The introduction of nanotechnology in the field of regenerative procedures has overcome these issues and demonstrated promising results. Nanoparticles can be used to deliver antibiotics at very low doses owing to their small size, thereby enhancing antimicrobial activity and reducing the cytotoxic effect. Additionally, nanofibrous scaffolds provide an environment that is favorable for stem-cell migration and proliferation, thereby favoring the regeneration of the pulp-dentin complex. Nanotechnology can be used in the construction of nanofibrous scaffolds incorporated with different bioactive nanoparticles for favorable clinical outcomes. Nonetheless, the role of nanotechnology and the controlled release of various bioactive nanomolecules enhancing stem cell proliferation and regeneration of true pulp-dentin complex remains poorly understood. Given the importance of nanotechnology in tissue regeneration, this review provides an overview of the potential applications of nanotechnology in tooth pulp-dentin regeneration.

## Introduction and background

A newly erupted immature permanent tooth with incomplete root apex formation usually takes a few years for complete root development after the tooth emerges in the mouth. Pulpal necrosis due to dental caries or traumatic injuries of immature permanent teeth can often affect root end development. Post-emergent root development consists of an increase in root length and root wall thickness as well as a narrowing of root canals in the apical region. A wide range of treatment protocols has previously been suggested to treat immature permanent teeth with necrotic pulps [1,2]. The apexification procedure is commonly used to treat immature necrotic permanent teeth with open root apices to induce the formation of the hard tissue barrier at the root apex. However, tissue vitality and root maturation along with apical closure cannot be achieved by this procedure. Consequently, the regenerative endodontic procedure, which aims to biologically and functionally regenerate the pulp-dentin complex in immature permanent teeth damaged by infection or trauma, was introduced by Nygaard-Ostby et al. in the 1960s [1]. This procedure involves crucial steps, such as disinfection of the root canal space, control of inflammation, and pulp tissue regeneration, to obtain the desired outcomes, which include complete tooth root maturation with signs of neurogenesis and resolution of the clinical signs and symptoms. However, true regeneration of the pulp-dentin complex is difficult to achieve, and factors such as the stage of root development, severity and duration of apical periodontitis, follow-up time, and trauma influence the treatment outcomes [3]. Several studies have been conducted in the field of regenerative endodontic procedures based on biological tissue engineering concepts that involve the triad of stem cells, scaffolds, and signaling molecules [1].

Various stem cells, such as dental pulp stem cells (DPSC) from permanent teeth, stem cells from the apical papilla (SCAP), immature dental stem cells from primary teeth, stem cells of human exfoliated teeth (SHED), and periodontal ligament stem cells (PDLSC), have been investigated for pulp-dentin regeneration [4,5]. The key element in regenerative endodontics is the proper disinfection of the canal system without damage to the endogenous stem cells in the apical papilla. The stem cells are introduced into the root canal space from the periapical region by inducing intracanal bleeding. Huang et al. used human DPSCs and SCAP, synthetic scaffolds, and human root segments to demonstrate the regeneration of pulp-like tissue and the formation of dentin-like mineral structures in immunocompromised mice [6].

Bioactive molecules are signaling molecules that initiate and maintain cellular interactions and aid in the regeneration of injured pulp tissues. They require a favorable microenvironment and a proper scaffold to aid in tissue regeneration. Scaffolds are designed to resemble the structure and complexity of the extracellular matrix (ECM) in the pulp. They provide mechanical support, promote cell adhesion, and enhance the growth and differentiation of stem cells. Scaffolds are biocompatible and can act as carriers of bioactive molecules and undergo biodegradation over time. Scaffolds can be classified as natural (collagen, fibrin, blood clot, platelet-rich fibrin, chitosan, silk, alginate, hyaluronic acid) or synthetic (polylactic acid, polyglycolic acid, polylactic-polyglycolic acid, polycaprolactone, poly (L-lactic acid)) based on the material used [7]. In a study conducted by Gangolli et al., DPSCs seeded onto a bi-layered poly (lactic-co-glycolic acid) scaffold fabricated with different pore diameters on each side penetrated through the entire thickness of the scaffold on the side with the large pores, whereas limited penetration was observed on the side with small pores. Nonetheless, penetration and differential odontoblastic differentiation of the DPSCs was observed on both sides of the scaffold [8].

Nanotechnology allows for the construction of nanofibrous scaffolds incorporated with different bioactive nanoparticles that possess the mechanical strength and properties required for the controlled release of bioactive agents. Owing to the unique characteristics of nanoparticles, which include their nano size, high surface area-to-volume ratio, enhanced solubility, heightened antibacterial activity, increased reactivity, and capacity to be functionalized with other reactive compounds, their use in regenerative endodontics has gained significant interest. Polymeric nanoparticles are used during the different stages of regenerative endodontics for dentin and pulp connective tissue formation, revascularization, re-innervation, and root end completion [9].

Various derivatives of nanoscale technology, including nanofibers, nanoshells, and nanotubes, have been used in endodontic tissue regeneration. Although several in vitro and in vivo studies on pulp-dentin regeneration have been conducted, the role of nanoparticles in the controlled release of various loaded bioactive molecules and the enhancement of stem cell proliferation remains to be understood for the proper application of nanotechnology in endodontic regeneration. This review outlines the role of nanomaterials in different areas of regenerative endodontics.

## Review

Methods

A review of the literature was performed by an electronic search of PubMed and Google Scholar for publications related to the evaluation of pulp-dentin tissue regeneration. The literature search was done using the keywords “nanoparticles,” “polymeric nanoparticles,” “nanofibers,” and “regenerative medicine.” Articles were chosen using the following inclusion criteria: articles published in the English language over the last 20 years, as well as studies related to the role of nanomaterials in regenerative endodontics. A total of 59 articles related to the field of endodontics and studying the role of tissue regeneration were considered. Articles related to the role of nanotechnology in other dental treatment procedures were not considered.

Nanoparticles and their role in endodontic regeneration

**Figure 1:**
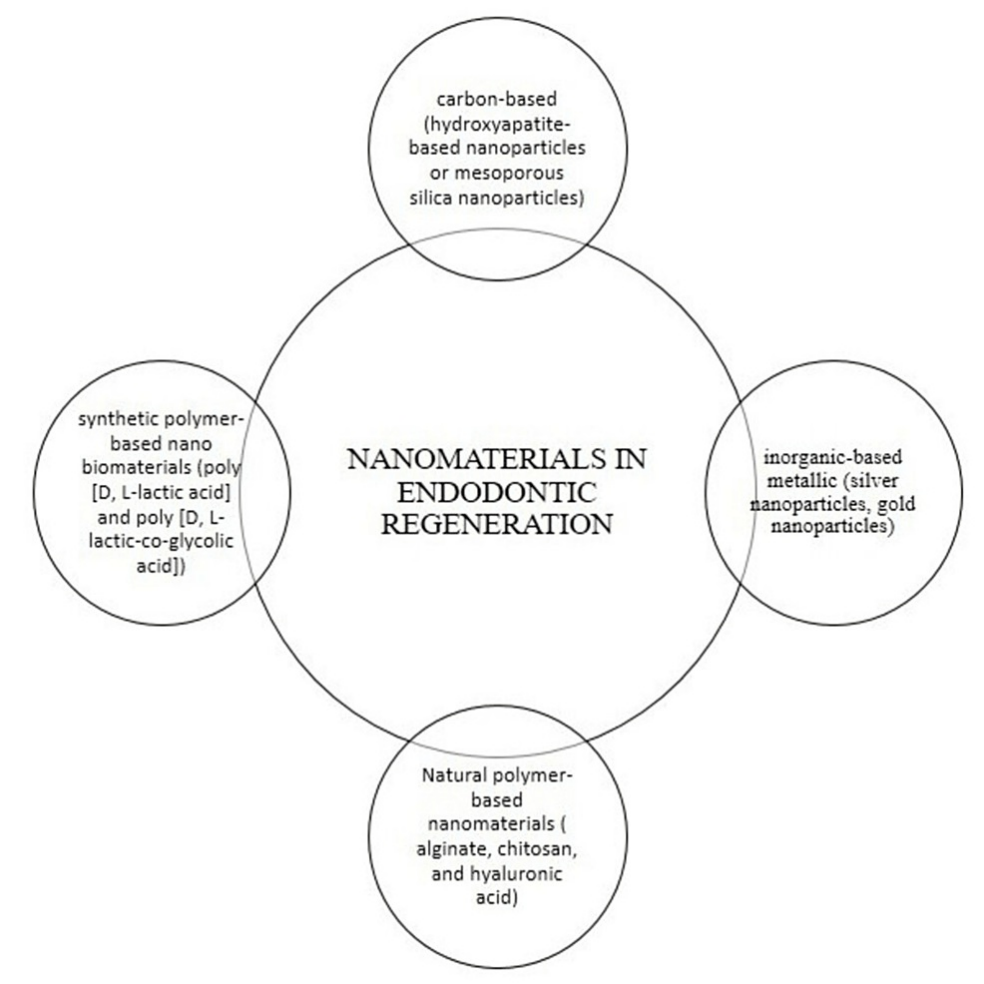
Schematic representation of nanoparticles in regenerative endodontics. Authors’ own work.

Nanoparticles and other products of nanoscale technology, such as nanofibers and nanopatterned surfaces, are employed in regenerative procedures. They are widely used for various functions such as drug delivery, gene delivery, antimicrobial action, and tissue regeneration. Based on the type of material used, nanoparticles can be classified as follows: polymeric-based (natural or synthetic polymer-based nanoparticles), inorganic-based metallic (silver nanoparticles, gold nanoparticles), carbon-based (hydroxyapatite-based nanoparticles or mesoporous silica nanoparticles), and biological protein peptide-based. Figure 1 represents the various types of nanomaterials used in endodontic regeneration.

Metallic nanoparticles such as silver nanoparticles have superior antimicrobial and anti-inflammatory properties and can be used to combat infections at much lower doses. Natural polymer-based nanomaterials (such as alginate, chitosan, and hyaluronic acid) are highly biocompatible and beneficial for cell adhesion and tissue regeneration but have low mechanical properties, which can result in the instability of the polymer-based nanobiomaterial leading to a burst release of the drug [10]. In contrast, synthetic polymer-based nanobiomaterials (such as poly (D, L-lactic acid) and poly (D, L-lactic-co-glycolic acid)) have greater mechanical strength and structural integrity. Thus, both natural and synthetic polymer-based nanobiomaterials can be used together to meet biological and physical requirements. By modifying the hollow interior microstructures, mesoporous materials can be used as carriers with adjustable release kinetics. Inorganic mesoporous silica nanoparticles are widely used in regenerative medicine for drug and gene delivery owing to their good biocompatibility and highly porous framework [11].

The presentation and local delivery of bioactive agents (such as growth factors, chemokines, and cytokines) in a controlled manner are essential for tissue engineering. Investigations on the use of polymeric nanoparticles as a carrier system for bioactive molecules have shown promising biomechanics and sustained release potential, thereby favoring tissue regeneration [12]. Incorporating bioactive materials, such as drugs or proteins, into a biodegradable nanopolymer increases the efficacy, bioavailability, and target tissue delivery potential of the bioactive molecule. Nanospheres (matrix system) and nanocapsules (reservoir system) are the two main types of polymeric nanoparticles used as drug carriers [12]. Nanocapsules consist of an inner oily core, where the drug is dissolved, and an outer polymeric shell, which controls the release profile of the drug. On the other hand, nanospheres are composed of continuous polymeric networks, wherein the drug is dissolved within the polymeric matrix or adsorbed onto the polymer [13].

Bioactive molecules can be incorporated into polymeric nanoparticles via chemical conjugation or physical encapsulation. A bioactive substance is enclosed within an inert material by the process of nanoencapsulation, which increases the stability and rate of release of bioactive substances at physiologically active sites [14]. For instance, core-shell structured lipid nanoparticles facilitate the encapsulation of hydrophobic drugs and exhibit a sustained drug release potential [15]. Lipid nanoformulations encapsulate hydrophilic and lipophilic regenerative agents that improve their bioavailability. Lipid nanoparticles may be considered alternative carriers for numerous other colloidal drug delivery systems owing to their size (50-1000 nm), the lack of need for an organic solvent, and the economic fabrication processes [16].

Nanofibers evaluated in endodontic regeneration

Nanofibers serve as nanoscale carriers for bioactive molecules and have been used during the various stages of regenerative endodontics. When used as a scaffold, the flexible nanofiber matrix meets several requirements, including those for three-dimensional (3D) matrix creation, drug delivery, attachment of growth factors, and adhesion of bioactive molecules, which encourages cell homing behavior for the regenerative process [17]. The surface texture, composition, network structure (random or aligned), and molecular orientation of the nanofiber must be optimized to improve its bioactivity. Nanofibers are available in different morphological forms, such as uniaxially aligned, biaxially oriented, porous fibers, ribbon-shaped, necklace-like, nanowebs, hollow tubes, and nanowire-in-microtubes [18]. A nanofibrous architecture enhances the adsorption of the cell adhesion protein when compared to a smooth-walled architecture scaffold. The nanofibrous architecture of poly (L) lactic acid scaffold is similar to the physical architecture of natural collagen fibers. It provides a favorable extracellular matrix-like environment for the attachment and proliferation of human DPSCs [19]. Nanofibers, used for the seeding of regenerative cells (such as dental pulp cells, mesenchymal cells, odontoblasts, and growth factors), act as a scaffold and provide a porous 3D surface that promotes the attachment of cells. Polymeric nanofibers can be fabricated by various methods such as self-assembly, electrospinning, and thermal-induced phase separation. These methods can be utilized to create 3D nanofibrous scaffolds with linked pore architectures that enable nutrition delivery, cell infiltration, and proliferation [20].

**Figure 2:**
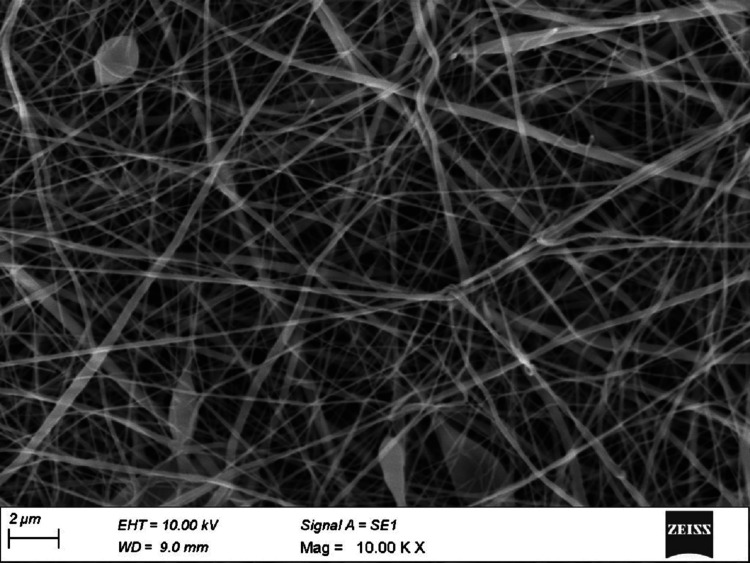
Scanning electron microscope observation: the electrospun nanofibrous poly(vinyl alcohol)/chitosan network membrane. Authors’ own work.

Electrospinning technology is commonly used to fabricate highly porous nanofibers with structures and mechanical properties similar to the extracellular matrix of native tissues. The image of an electrospun poly(vinyl alcohol) (PVA)/chitosan nanofiber mimicking the extracellular matrix is shown in Figure 2.

The electrospun nanofiber can be used as a carrier of active molecules, and this functionalization of nanofibers can be performed using various techniques such as surface graft polymerization and wet chemical treatment [21]. Antibiotics incorporated into electrospun nanofibrous membranes can exhibit more pronounced disinfection at a relatively lower dosage, thereby minimizing the adverse toxic effects of currently used high-concentration antibiotics. Studies have shown that electrospinning does not jeopardize the therapeutic properties of the incorporated material [22]. A nanopolycaprolactone-gelatin matrix incorporated with mesoporous bioactive nanoparticles can enhance the odontogenic differentiation of human DPSCs.

Low-dose simvastatin incorporated in a poly (L) lactic acid nano scaffold was reported to promote dentin regeneration by reducing inflammatory reactions and increasing stem cell regenerative capacity in inflamed dental pulp tissue [23]. Scaffold-mediated angiogenesis from the surrounding tissue is desirable. Moreover, the presence of blood vessels inside the scaffold improves the supply of nutrients/oxygen and the removal of metabolic waste, thereby creating a favorable environment for the growth of DPSCs. Endothelial cells cultured on simvastatin/nanofibrous-poly (L) lactic acid scaffolds are reported to form highly connected vascular-like structures, which demonstrates the angiogenesis-promoting properties of dental pulp cells [23]. Eap et al. reported that nanofibrous polycaprolactone scaffolds functionalized by a neural growth factor can induce pulp innervation and exhibit signs of tissue homeostasis [24]. In another study, dentin regeneration by human pulp stem cells is enhanced using 3D nanofibrous gelatin/magnesium phosphate scaffolds, resulting in a controlled release of metallic ions [25]. An injectable scaffold is more beneficial than an implantable bulk scaffold for dental regenerative procedures. Kuang et al. used poly (L) lactic acid-block-poly (L-lysine) copolymers to fabricate nanofibrous spongy microspheres as an injectable cell carrier by employing self-assembly and thermally induced phase separation techniques [26]. The nanofibrous spongy microspheres with interconnected pores improve the proliferation and odontogenic differentiation of human DPSCs compared to the nanofibrous microspheres without pore structures, thus serving as a potential injectable cell carrier system for dentin regeneration. In another study, the vascular endothelial growth factor is bonded with heparin and encapsulated into gelatin nanospheres, which are then entrapped within nanofibrous-poly (L) lactic acid microspheres and used as injectable growth factor carriers. Successful regeneration of pulp-like tissue, which filled the entire root canal space, is observed [27]. Table 1 shows the different nanofibers evaluated in the field of regenerative endodontics.

**Table 1:** Role of different nanofibers used during the various stages of endodontic regeneration.

Nanofiber	Targeted tissue	Result
Poly (L-lactic acid) (PLLA) nanoscaffold loaded with low-dose simvastatin [23]	Dental pulp stem cells	Increased stem cell regenerative capacity. Good angiogenic promoting property of dental pulp cells
Polycaprolactone scaffolds [24]	Neural growth factor	Innervation of the pulp
Nanofibrous gelatin/magnesium phosphate scaffolds [25]	Human pulp stem cells	Dentin regeneration
Poly (L-lactic acid)-block-poly (L-lysine) nanofiber [26]	Human dental pulp stem cells	Enhanced proliferation and odontogenic differentiation
Nanofibrous, poly (L-lactic acid) microspheres [27]	Vascular endothelial growth factor	Pulp-like tissues regeneration in entire root canal space
Polycaprolactone/gelatin and nano-hydroxyapatite composite scaffold [28]	Dental pulp stem cells	Enhanced proliferation and odontogenic differentiation
Apatite-mineralized polycaprolactone nanofibrous scaffold [29]	Human dental pulp cells	Enhanced odontogenic differentiation
Composite biopolymer nanofiber loaded with bioactive glass nanoparticles [30]	Human dental pulp cells	Promoted odontogenic differentiation
Triple antibiotic-eluting nanofiber [31,32]	Multispecies biofilm cytotoxic effect on stem cell	Better antibacterial action at low concentration, and no significant cytotoxic effect
Carboxymethyl chitosan-based scaffold [33]	Transforming growth factor-β1	Enhanced differentiation and migration of stem cells
Alginate/nano-hydroxyapatite scaffold [34]	Dental pulp stem cells	Enhanced osteogenic differentiation. Promoted calcium deposition and biomineralization

Different materials used in endodontic tissue regeneration in nanoscale form

Chitosan

Chitosan nanoparticles have shown promising results in the local delivery of bioactive molecules due to their nano size, large surface area, increased chemical reactivity, and potential to be conjugated with other molecules [35]. It is a bioactive biopolymer with alterable porosity to permit the transport of nutrients, biomolecules, and toxic cellular products. The complexation of recombinant keratinocyte growth factor with mucoadhesive low-molecular-weight chitosan is reported to enhance the stability and biological function of the growth factor [36]. Likewise, a carboxymethyl chitosan-based scaffold incorporated with transforming growth factor (TGF)-β1-loaded chitosan nanoparticles are found to enhance the differentiation and migration of stem cells [33]. In another study, alkaline phosphatase, an early marker of odontoblast-like differentiation, exhibited improved activity in SCAP due to the slow and continuous release of bovine serum albumin from chitosan nanoparticles [37]. Shrestha et al. reported that the odontogenic stimulant dexamethasone improves the odontogenic differentiation of SCAP when loaded onto chitosan nanoparticles [38]. The harmful effects of using sodium hypochlorite as an endodontic irrigant are found to be reduced when the dentin surface is conditioned with either chitosan nanoparticles or dexamethasone-modified chitosan nanoparticles; conditioning with chitosan nanoparticles stimulated the adherence, viability, and differentiation of SCAP [39].

Hydroxyapatite

Biodegradable synthetic polymers such as polycaprolactone, polyglycolide, and polylactide exhibit inadequate cell adherence and proliferation due to the lack of biological recognition sites. These polymer material scaffolds can be supplemented by hydroxyapatite crystals, which provide inorganic reinforcement and simulate the natural dentin extracellular matrix. A previous study showed that the addition of nanohydroxyapatite to polymer scaffolds enhances the cellular adherence and proliferation of osteoblast-like cells [40]. Nanohydroxyapatite increases protein absorption, cell adhesion, and cell proliferation; additionally, it has a high solubility, which promotes an increase in the bioactivity of the polymer. Alginate can be fabricated into 3D porous biodegradable networks. DPSCs seeded on alginate and nanohydroxyapatite scaffold are found to express osteogenic differentiation-related markers and promote calcium deposition and biomineralization [34]. Electrospun composite scaffolds made of polycaprolactone/gelatin and nanohydroxyapatite enhanced the proliferation and odontogenic differentiation of DPSCs [28]. According to Kim et al., apatite-mineralized polycaprolactone nanofibrous scaffolds favor the development and odontogenic differentiation of human dental pulp cells via the integrin-mediated signaling pathway when compared to the plain polycaprolactone fibers [29].

Cellulose Nanocrystals

A scaffold architecture with high porosity and interconnected pores allowing for adequate nutrient diffusion, cell migration, and vascularization may have poor mechanical properties. Silva et al. reported that injectable hyaluronic acid hydrogels reinforced with cellulose nanocrystals have higher scaffold network stiffness and stability due to their action as effective junctional elements, thereby favoring cell adhesion, migration, and proliferation [41]. The incorporation of a platelet lysate, which is rich in proangiogenic and chemotactic factors, into reinforced hydrogel enhanced the revascularization and regeneration of pulpal tissue [41].

Bioactive Glass

Lim et al. incorporated dexamethasone molecules loaded with bioactive glass nanoparticles within the biopolymer nanofiber matrix through electrospinning and found that this nanofiber composite exhibits a sustained, controlled release of dexamethasone for 14 days. Additionally, it enhances the odontogenic differentiation of human dental pulp cells via the activation of integrins, bone morphogenetic protein, and the mammalian target of rapamycin (mTOR) signaling pathway [30]. The mechanical properties of scaffolds can be improved by the incorporation of bioactive glass nanoparticles. These nanoparticles have better biocompatibility, promote cell adhesion and migration, and demonstrate stronger osteogenic abilities than microscale bioactive glass. Kim et al. showed that a composite nanofibrous scaffold of polycaprolactone/gelatin and mesoporous bioactive glass nanoparticles promoted the odontogenic differentiation of human dental pulp cells by activating integrins, bone morphogenic proteins (BMP), and the mitogen-activated protein kinase signaling pathway [42]. Bae et al. reported similar results, wherein the nanocomposite collagen/nano bioglass scaffold matrix induced the growth and odontogenic differentiation of stem cells more effectively than collagen alone [43].

Metal Ions

Biomaterials that promote osteogenic and angiogenic activity and exhibit antimicrobial potential are ideally chosen for regenerative procedures. Metal ions such as magnesium, strontium, zinc, and copper ions are involved in tissue development and repair. Graphene oxide copper nanocomposite has been shown to enhance the odontogenic and neurovascularization activities of DPSCs [44]. Low-concentration copper ions have antimicrobial, osteogenic, and angiogenic effects when administered in controlled-releases dosage form [45]. El-Fiqi et al. evaluated the use of a mesoporous bioactive glass nano-delivery system incorporating silicate, calcium, copper, and epidermal growth factors and report excellent antibacterial, angiogenic, and odontogenic effects [46]. The copper ions and epidermal growth factors released from glass nanospheres suppressed the growth of *Enterococcus faecalis* and stimulate the angiogenic potential of endothelial cells.

Calcium Silicate Nanoparticles

Calcium silicate-based materials promote hard tissue regeneration and stimulate odontogenic and osteogenic differentiation in various types of cells. Huang et al. fabricated an injectable paste of mesoporous calcium silicate nanoparticles with organized mesoporous channels that exhibited high surface areas and pore volumes. These nanoparticles demonstrated a sustained release pattern of calcium and silicate ions, thus indicating their potential as a novel material in regenerative endodontics [47]. In another study, a unique, ready-to-use calcium silicate-based nanoparticulate bioceramic paste demonstrates excellent apatite formation; enhances the adhesion, migration, and attachment of DPSCs; and induces the formation of a dentin bridge for pulp repair [48].

Gold Nanoparticles

To study and visualize the interactions between stem cells and scaffolds and to know how the cells are spatially distributed after seeding, Biz et al. combined gold nanoparticles with poly-L-lysine and incorporated them into dental pulp stem cells as gold nanoparticles have a higher coefficient of X-ray absorption and provide a superior contrast. The authors stated that an increase in cell radiopacity allows for the identification of viable cell distribution in scaffolds, and the functions of the stem cells remained unaffected [49]. A previous study has shown that gold nanoparticle-incorporated calcium phosphate cement significantly enhanced the osteogenic functions of hDPSCs [50]. In another study, gold nanoparticles are reported to inhibit pathogenic *Candida* *albicans* biofilm formation and invasion into DPSCs [51].

Antimicrobials

Sodium hypochlorite irrigation of the root canal system and the use of double or triple antibiotic paste (TAP) can result in maximum bacterial eradication, but when used in high concentrations, they can negatively impact the survival of dental-derived stem cells [52]. To overcome this cytotoxic challenge, novel antibiotic-containing polymer nanofibers are developed as a 3D tubular drug delivery construct that can be placed inside the root canals of the necrotic teeth [53]. This allows for the use of antibiotics at lower concentrations with a sustained release rate, which facilitates regeneration. Antimicrobial nanoparticles can infiltrate bacterial biofilms and interact electrostatically with bacterial cell walls leading to cell membrane disruption, increased cell permeability, protein breakdown, and DNA damage, ultimately resulting in cell death.

TAP, which contains metronidazole, ciprofloxacin, and minocycline, has been recommended for canal disinfection during regenerative procedures. However, the drawbacks of TAP include notable tooth discoloration and significant dental stem cell death when used at considerably high concentrations. Albuquerque et al. reported that the antimicrobial properties of 3D tubular-shaped triple antibiotic-eluting nanofiber constructs containing significantly low concentrations of metronidazole, ciprofloxacin, and minocycline against multispecies biofilm are similar to those observed with high concentrations of TAP [31]. Similar results were also reported by Palasuk et al. wherein nanocomposite scaffolds containing metronidazole or ciprofloxacin and polydioxanone exhibited good antimicrobial activity against *Enterococcus faecalis* and *Porphyromonas gingivalis* with no toxic effects on human DPSCs [53]. The kinetics of antibiotic release from polydioxanone nanofibers in the work by Kamocki et al. shows an initial burst release, followed by a sustained release for up to 14 days, providing a long-lasting antibacterial impact [54]. In a recent study, clindamycin-modified triple antibiotic (minocycline-free) polydioxanone (PDS) polymer nanofibers exhibit significant antimicrobial effects and low cytotoxicity. Scanning electron microscopy shows that clindamycin-containing nanofibers have smaller diameters than antibiotic-free PDS nanofibers, providing more surface area for drug release over time [32]. Silver nanoparticles exert significant antibacterial effects at low concentrations [55]. Compared to other micro- or macro-sized materials, nanoscale materials are more substantial and can penetrate the dentinal tubules and lateral canals with ease [56]. Additionally, oxidative stress and mitochondrial dysfunction can be brought on by silver nanoparticles in human cells. Alternatively, low concentrations of silver nanoparticles do not appear to be detrimental to mammalian cells, and the green synthesis of low concentrations of silver nanoparticles does not affect human dermal fibroblasts in a recent study by Oncu et al. [57]. In another study using DPSCs, carboxymethyl cellulose-silver nanoparticles exhibit good antimicrobial properties and low toxicity at a concentration of 16%; however, cytotoxic effects are observed at concentrations of >50% [58].

Biodegradable poly (D, L) lactic acid nanoparticles, which have low immunogenicity, can be used to deliver antifungal and antibacterial drugs. Bekhouche et al. evaluated the antibacterial efficacy of clindamycin-loaded poly (D, L) lactic acid nanoparticles incorporated into fibrin hydrogel and reported better antibacterial and antibiofilm activities without any effect on the cell viability, thereby creating an aseptic environment that is favorable for tissue regeneration [59].

## Conclusions

Nanodentistry is the application of nanotechnology for diagnosing, treating, and preventing oral and dental diseases. Nanotechnology has demonstrated promise as a carrier of bioactive molecules and for the controlled delivery of drugs and growth factors. In this review, we have addressed the research conducted in the field of nanotechnology that can be used in the fabrication of various functionalized nanoparticles for their use in regenerative endodontics. These functionalized nanoparticles can be employed as novel materials to enhance antimicrobial drug activity at a relatively smaller dose and improve regenerative outcomes. The combination of stem cells and bioactive nanostructured scaffolds creates a better environment for the reconstruction of the dental pulp. Within the limitations of this review which does not include a literature search that considers the chances of potential adverse effects of nanomaterial use in regenerative endodontics, it can be stated that studies on long-term clinical trials are needed to evaluate the successful clinical application of nanomaterials in regenerative endodontics.
